# Gallbladder atrophy associated with pancreatitis: Clinical and advanced imaging diagnosis in a dog

**DOI:** 10.17221/76/2023-VETMED

**Published:** 2023-11-20

**Authors:** Donghyun Han, Dong-In Jung

**Affiliations:** ^1^Choi Youngmin Animal Medical Center, Seoul, Republic of Korea; ^2^Institute of Animal Medicine, College of Veterinary Medicine, Gyeongsang National University, Jinju, Republic of Korea

**Keywords:** cholecystitis, cholelithiasis, computed tomography, gastroinetstinal symptom, nodule

## Abstract

Gallbladder atrophy (GBA) is characterised by a reduction in the size and volume of the gallbladder. In human medicine, it is well-established that GBA frequently occurs together with pathologies affecting the gallbladder and pancreas. However, to the best of our knowledge, there is currently a dearth of reported cases of GBA in dogs within the veterinary field. In this study, we present a case report of GBA in a 7-year-old Yorkshire Terrier. The diagnosis of GBA was confirmed using abdominal ultrasonography and advanced imaging techniques, including computed tomography, which were performed over a 4-year period. The patient initially presented with predominantly gastrointestinal symptoms, which were subsequently diagnosed and treated as pancreatitis. Concurrently, a gallbladder nodule and an anomalous structure suspected to be cholelithiasis were identified. However, during the 4-year follow-up, the gallbladder structure regressed, leaving only the presence of the gallbladder nodule. Notably, cholecystectomy was not performed, and apart from pancreatitis-related symptoms, the patient did not show any gallbladder-related problems throughout the spontaneous atrophic process. Based on these findings, we propose that the observed GBA was likely induced by cholecystitis associated with pancreatitis. This case underscores the significance of considering GBA as a potential diagnosis in canine patients presenting with pancreatitis and gastrointestinal symptoms. Furthermore, it highlights the value of comprehensive diagnostic imaging in accurately determining the underlying cause of these symptoms.

Gallbladder atrophy (GBA) refers to the condition characterised by the reduction in size and volume of the gallbladder. In humans, GBA commonly occurs along with cholecystitis (Shirah et al. 2017), cholelithiasis (Shirah et al. 2017), micro-gallbladder (MG) (Kramer et al. 1983), gallbladder volvulus (GV) (Luo et al. 2014), and pancreatitis (Jiang et al. 2023) and is frequently incidentally diagnosed on abdominal ultrasonography (Maldonado et al. 2014). Furthermore, microscopic GBA has been reported in humans with cystic fibrosis (CF) (King et al. 2000).

In patients with chronic gallbladder conditions such as cholecystitis or cholelithiasis, a prolonged gallbladder disease can lead to increased pancreatic secretion, resulting in pancreatic injury and an elevated risk of pancreatitis (Jiang et al. 2023). Pancreatitis, characterised by inflammation of the pancreas, can lead to secondary effects on the adjacent gallbladder. The exact mechanisms underlying GBA in pancreatitis are not fully understood; however, it is believed to be a consequence of the inflammatory response and alterations in bile flow dynamics. In cases of pancreatitis, the inflammatory process can extend to the gallbladder, thereby resulting in structural changes and functional impairment. This can manifest itself as gallbladder wall thinning, decreased contractility, and ultimately, GBA.

This case report presents a unique occurrence of GBA with pancreatitis in a 7-year-old Yorkshire Terrier exhibiting gastrointestinal symptoms. The initial imaging examination revealed evidence of pancreatitis and the presence of a gallbladder nodule, which remained consistent during a follow-up visit after 4 years. However, the subsequent examination demonstrated GBA, making its confirmation unattainable. To our knowledge, there have been no case reports of GBA in dogs.

This case highlights the significance of considering GBA as a potential diagnosis in dogs presenting with gastrointestinal symptoms and emphasises the value of thorough diagnostic imaging to accurately identify the underlying cause of these symptoms.

## Case description

**Figure 1:**
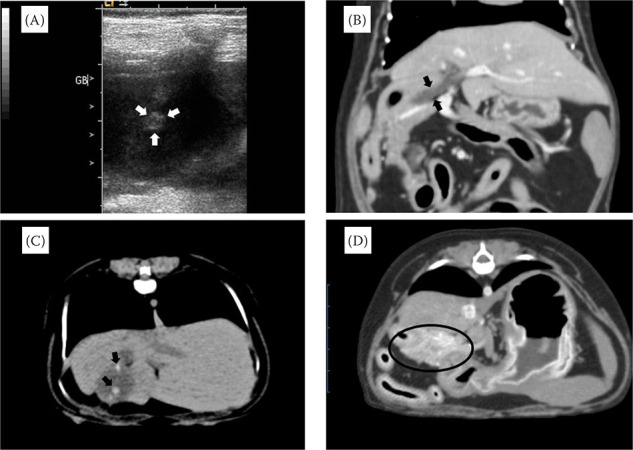
Specific findings of the gallbladder and pancreas obtained from imaging of the dog (A) Abdominal ultrasonography showing a hyperechoic nodule (white arrows) suspected to be cholecystitis in the bile with a hyperechoic texture. (B) Visualisation of the dilated common bile duct (black arrows) on post-contrast reconstructed coronal images of abdominal computed tomography (CT). (C) Hyperattenuated mineralised foci (indicated by black arrows) are observed in the hypoattenuated gallbladder on pre-contrast axial images of abdominal CT. (D) The pancreas (indicated by the black ellipse) is depicted in the post-contrast image with heterogeneous contrast enhancement on abdominal CT

A 7-year-old Yorkshire Terrier was presented to the private veterinary hospital with chief complaints of vomiting, anorexia, and abdominal discomfort for a few days. The dog weighed 3.2 kg with a body condition score of 4 of 9 points. Physical examination results showed no jaundice; however, mild dehydration and discomfort on abdominal palpation were observed. Complete blood count (CBC) results showed a mild neutrophilic leukocytosis (Table 1). In serum chemistry tests, it was confirmed that the liver-related indicators of alanine aminotransferase [ALT, 5.46 μkat/l (reference range: 0.17–1.96 μkat/l)], aspartate aminotransferase [AST, 1.05 μkat/l (reference range: 0.23–0.75 μkat/l)], gammaglutamyl transferase [GGT, 0.25 μkat/l (reference range: 0–0.12 μkat/l)], and total bilirubin [20.52 μmol/l (reference range: 1.71–10.26 μmol/l)] were higher than normal. Additionally, it was confirmed that the cholesterol level [13.78 mmol/l (reference range: 3.24–6.99 mmol/l)] increased and the blood urea nitrogen [BUN, 2.14 mmol/l (reference range: 2.50–8.92 mmol/l)] decreased. The activities of amylase [33.15 μkat/l (reference range: 3.34–20.04 μkat/l)] and lipase [38.43 μkat/l (reference range: 3.34–30.06 μkat/l)] were also elevated. Moreover, a canine pancreas-specific lipase [cPL, 1 928 μg/l (reference range: < 400 μg/l)] test with high specificity of the pancreas was performed, and the test result showed pancreatitis. Several abdominal ultrasonography examinations in a modified transverse plane, with the patient in ventro-dorsal position at the right upper abdomen, revealed no gallbladder wall thickening; however, a small hyperechoic atypical nodule, which was suspected to be a gallstone, was identified (Figure 1A). Computed tomography (CT) was performed on the dog in a dorso-ventral/sternal recumbency to obtain more precise images and confirm the structural condition of the biliary tract. CT (Asteion 4^®^; Toshiba, Tokyo, Japan; Ian Animal Diagnostic Imaging Center, Seoul, Republic of Korea) examinations revealed a dilated common bile duct (Figure 1B) and mineralised foci, which were presumed to be cholelithiasis (Figure 1C). Those that did not follow the direction of gravity were assumed to be stones in the form of adhesions to the gallbladder wall. Additionally, the pancreatic body was observed in the post-contrast image with heterogeneous contrast enhancement on abdominal CT (Figure 1D). Based on this medical information, the dog was diagnosed with pancreatitis along with suspected cholelithiasis and cholestasis.

**Table 1:** Haematological parameters of the patient in this case at the time of the initial visit

Parameter	Result	Reference ranges
Haematocrit (× 10^–2^ l/l)	45.3	35.0–57.0
Haemoglobin (× 10 g/l)	15.2	11.9–18.9
RBCs (× 10^12^/l)	6.64	4.95–7.87
MCV (fl)	68	66–77
MCH (pg)	22.9	21.0–26.2
MCHC (× 10 g/l)	33.5	32.0–36.3
MPV (fl)	10.0	6.1–10.1
WBCs (× 10^9^/l)	15.8	5.0–14.1
Neutrophils (× 10^9^/l)	13.2	2.9–12.0
Eosinophils (× 10^9^/l)	0.5	0.0–1.3
Lymphocytes (× 10^9^/l)	1.9	0.4–2.9
Monocytes (× 10^9^/l)	0.2	0.0–0.9
Platelets (× 10^9^/l)	125	211–621

MCH = mean corpuscular haemoglobin; MCHC = mean corpuscular haemoglobin concentration; MCV = mean corpuscular volume; MPV = mean platelet volume; RBCs = red blood cells; WBCs = white blood cells

The dog was hospitalised for the treatment of pancreatitis and hepatic dysfunction due to cholestasis. Intensive fluid therapy was administered, and food was restricted. Liver dysfunction with cholecystitis was suspected on the basis of blood tests and imaging examination results, and cholelithiasis was most strongly suspected as the cause. For cholelithiasis, cholecystectomy was considered; however, it was excluded because pancreatitis in the current patient was a priority treatment, and consultation with the dog’s owner led to the decision to not proceed with the surgery. In addition, metronidazole (20 mg/kg i.v. and b.i.d.; Flagyl Inj^®^; Alvogen Korea Co., Ltd., Seoul, Republic of Korea) was administered as an antibiotic to treat bacterial overgrowth associated with cholelithiasis and cholestasis, maropitant (1 mg/kg s.c. and s.i.d.; Cerenia^®^, Gerona, Spain) was administered as an antiemetic prescribed for vomiting, and gabexate mesylate (i.v. infusion at a dose of 1 mg/kg/h; Foy^®^, Donga ST Pharm., Seoul, Republic of Korea) as a synthetic protease inhibitor administered for reducing the mortality rate due to pancreatitis (Satoh et al. 2004). After 3 days of hospitalisation, the clinical signs of vomiting and anorexia were alleviated, and the dog was able to eat and drink voluntarily. Subsequently, ursodeoxycholic acid (10 mg/kg p.o. and b.i.d.; Daewoong Bio Ursodeoxycholic Acid Tab^®^; Daewoong Bio Inc., Hwasung, Republic of Korea) for reducing cholesterol secretion, *S*-adenosyl-l-methionine (10 mg/kg p.o., b.i.d.; Sameron Tab^®^; Shin Poong Pharm, Seoul, Republic of Korea) as an antioxidant for liver dysfunction, and a combination of pancreatic enzyme preparation for pancreatitis were prescribed. To assess the patient’s improvement, CBC and serum chemistry tests (ALT, AST, GGT, total bilirubin, cholesterol, BUN, amylase, lipase, and cPL) were conducted on multiple occasions to monitor the patient’s condition. Two weeks after initiating the treatment, the previously abnormal values returned to the physiological range.

**Figure 2:**
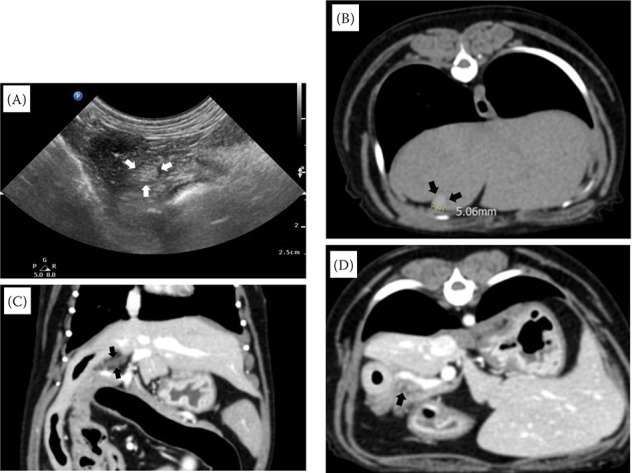
Specific findings of the gallbladder obtained during imaging tests at the 4-year follow up (A) Abdominal ultrasonography reveals the absence of a gallbladder containing bile, with visualisation of an atrophic hyperechoic nodule (white arrows). (B) A hyperattenuated atrophic gallbladder is observed in the pre-contrast axial image of abdominal CT. (C) The common bile duct (black arrows) measures 2.1 mm in diameter and opens normally into the duodenum, as shown in the post-contrast reconstructed coronal image of abdominal CT. (D) A small irregular cystic lesion (black arrow) is detected in the image of the pancreatic body on the post-contrast axial image of abdominal CT

Four years later, the same dog presented with similar symptoms of vomiting and anorexia. Based on the medical history provided by the patient’s owner over the past four years, it was confirmed that the patient experienced intermittent mild digestive symptoms. These symptoms exhibited self-limiting characteristics for brief periods, and the patient received intermittent treatment for pancreatitis at a local veterinary hospital. A basic physical examination was performed, and epigastric pain was confirmed; however, jaundice was not seen in the gingival mucosa. The CBC results showed mild neutrophilic leukocytosis (Table 2). Serum chemistry test results showed high amylase [28.79 μkat/l (reference range: 3.34–20.04 μkat/l)] and lipase activities [32.48 μkat/l (reference range: 3.34–30.06 μkat/l)]. Furthermore, a specific test for pancreatitis, the cPL [1 521 μg/l (reference range: < 400 μg/l)] test, was conducted and yielded a significantly elevated result. However, unlike before, the liver-related indicators appeared normal. Ultrasonography revealed abnormalities in the gallbladder structure, including a 5-mm hyperechoic nodule, exhibiting a similar size and appearance to the nodule detected 4 years ago (Figure 2A). The CT (Brivo 385 CT^®^; GE Healthcare, IL, USA; Helix Animal Medical Center, Seoul, Republic of Korea) examinations were also performed, and showed results similar to ultrasonography, with a traced gallbladder of approximately 5 mm in size (Figure 2B). The common bile duct measured 2.1 mm in diameter and opened normally into the duodenum (Figure 2C). Furthermore, a small irregular cystic lesion was visualised in the image of the pancreatic body (Figure 2D). Micro-gallbladder (MG), which is recommended to be disregarded if asymptomatic or not complaining of pain, could be suspected; however, it was excluded from the differential diagnosis because no cases of cystic fibrosis (CF) have been reported in dogs and cats. Additionally, gallbladder infarction (GBI) was considered (Olivares et al. 2018); however, the absence of peritonitis due to bile leakage and the patient’s history indicated the absence of specific symptoms associated with it over the 4-year period. GBA was suspected in the dog; however, no evidence of liver dysfunction was noted. Therefore, fluid treatment, maropitant (1 mg/kg s.c., s.i.d.+ Cerenia^®^; Zoetis, Gerona, Spain) as an antiemetic, and gabexate mesylate (i.v. infusion in a dose of 1 mg/kg/h; Foy^®^; Donga ST Pharm., Seoul, Republic of Korea) as a synthetic protease inhibitor were administered for the treatment of pancreatitis as before.

**Table 2:** Haematological parameters of the patient in this case at the time of 4 years after the initial visit

Parameter	Result	Reference ranges
Haematocrit (× 10^–2^ l/l)	52.9	35.0–57.0
Haemoglobin (× 10 g/l)	17.3	11.9–18.9
RBCs (× 10^12^/l)	7.71	4.95–7.87
MCV (fl)	69	66–77
MCH (pg)	32.8	21.0–26.2
MCHC (× 10 g/l)	33.5	32.0–36.3
MPV (fl)	8.9	6.1–10.1
WBCs (× 10^9^/l)	15.1	5.0–14.1
Neutrophils (× 10^9^/l)	12.2	2.9–12.0
Eosinophils (× 10^9^/l)	0.7	0.0–1.3
Lymphocytes (× 10^9^/l)	2.0	0.4–2.9
Monocytes (× 10^9^/l)	0.2	0.0–0.9
Platelets (× 10^9^/l)	226	211–621

MCH = mean corpuscular haemoglobin; MCHC = mean corpuscular haemoglobin concentration; MCV = mean corpuscular volume; MPV = mean platelet volume; RBCs = red blood cells; WBCs = white blood cells

After receiving the treatment, the patient’s symptoms resolved, and he was discharged. During the following three years, the dog intermittently experienced episodes of anorexia associated with the problem in the pancreas but otherwise maintained a normal quality of life.

## DISCUSSION

The gallbladder is a small pear-shaped sac located below the liver. It stores bile, a fluid produced by the liver that aids digestion. The gallbladder contracts and expels bile through the bile duct into the small intestine upon fatty food ingestion (Hardy et al. 2006). The physiological function of the gallbladder is to concentrate some of the bile produced by the liver and secrete it into the duodenum during meals to aid in lipid absorption (Hofmann 1999). In this process, when the bile is abnormally concentrated, it may turn into a hard gallstone. Several causes of cholelithiasis include the age, genetic factors, obesity, and excessive intake of cholesterol, fat, and high-calorie food (Pak and Lindseth 2016). As most stones contain insufficient calcium to be visible on radiographs and often have limited diagnostic value, cholelithiasis may go undetected (Aguirre 2017). Upon the initial CT of the patient, the presence of two hyperatteuated structures within the gallbladder raised suspicion of cholelithiasis. However, upon re-evaluation of the CT images after a 4-year interval, only one abnormal structure was identified within the gallbladder, whereas the normal gallbladder structure was no longer discernible. It can be speculated that the hyperatteuated nodule had potentially traversed the biliary tract and translocated into the digestive tract over the 4-year course. The remaining structure exhibited a similar size and location to the previous image; however, the temporal difference observed at the imaging site was sufficient to classify it as a distinct nodule rather than assuming it to be cholelithiasis. This differentiation can be attributed to the upgraded number of channels and image acquisition software implemented in the CT scanner used during the subsequent evaluation performed 4 years later.

As the liver is responsible for converting ammonia to urea, BUN levels may decrease secondary to liver disease (Lee et al. 2015). Impaired bile flow (cholestasis) causes increased alkaline phosphatase (ALP) and GGT synthesis (Siddique and Kowdley 2012). ALP is a sensitive indicator of cholestasis in dogs (Center et al. 1992). With cholestatic disorders, an increased ALP activity precedes hyperbilirubinemia (Taboada and Meyer 1989). ALT, a liver enzyme predominantly present in hepatocytes, leaks from the liver cells and increases in the bloodstream as a result of liver cell damage (McGill 2016). AST is primarily associated with the mitochondria but is also present in the cytoplasm. The release of AST from the mitochondria requires a severe insult. Thus, with hepatocyte injury, ALT is more readily released, and its activity will usually be higher than that of serum AST (Kim et al. 2008). Considering these points, it is strongly hypothesised that the dog, in this case, had cholestasis due to cholelithiasis, which led to liver dysfunction. Abnormal findings in blood tests are presumed to be changes due to bile stagnation. Moreover, cholestasis is believed to be a secondary change due to pancreatitis, and the biliary tract inflammation and hepatitis are presumed to be secondary. Pancreatitis and inflammation of the hepatobiliary system frequently occur concurrently in cats (triaditis), and it is presumed to be caused by ascending infection of the pancreas and hepatobiliary system, mainly due to inflammation of the gastrointestinal system (Simpson 2015). In the present case, the dog exhibited vomiting and anorexia, which led to a diagnosis of pancreatitis. Further imaging examinations revealed suspected cholelithiasis; however, no evidence of obstruction was observed. The dog was treated for pancreatitis and liver disease and eventually recovered. Four years later, the dog presented again with similar symptoms; however, this time, imaging examinations revealed suspected atrophy. In addition, no specific abnormalities were found in other liver-related serum chemistry test parameters, including ALP. In the patient in this case, gallbladder atrophy has occurred, preventing the storage of bile produced in the liver in the gallbladder. Therefore, it can be inferred that cholestatic disorders, such as the stagnation or obstruction of bile reaching the intestine through the common bile duct or reflux into the liver, did not occur.

GBA is a rare finding in humans. In veterinary medicine, there have been no reports of GBA in dogs or cats, making this case particularly noteworthy. Furthermore, atrophic gallbladders are observed in gallbladder carcinomas, which have been reported in humans (Hatakeyama et al. 2009). Despite the gap in the treatment period of this patient, no evidence of tumour was observed anywhere in the body, including the gallbladder, through clinical evidence observed 4 years after the first treatment. Therefore, tumours, including carcinomas, are not the cause of GBA in this case.

Gallbladder shrinkage is also observed in the MG, a widespread disorder that can lead to chronic inflammation or atrophy of the gallbladder due to CF-related liver disease (Kramer et al. 1983). CF is an autosomal recessive genetic disease involving the CF transmembrane conductance regulator protein (CFTR), which plays a significant role in regulating the secretory function and absorption in the respiratory, genital, and gastrointestinal tracts, including the liver and pancreas (Bartlett et al. 2009; Chen et al. 2010). In humans, bronchiectasis can be classified into CF-related bronchiectasis and non-CF bronchiectasis. A study in dogs reported that naturally occurring CFTR mutations are relatively common in domestic dogs; however, the study failed to ascertain a higher expression rate of CFTR mutations in dogs with bronchiectasis (Spadafora et al. 2010). Bronchiectasis in dogs and cats is probably primarily due to non-CF bronchiectasis. Non-CF aetiologies of bronchiectasis in both humans and dogs include congenital or inherited disorders. Considering this, it is reasonable to conclude that the GBA in this case is not CF-related MG.

GV, also known as a “floating gallbladder,” is a distinct condition from the one in this case where bile flow is not smooth, and the common bile duct appears normal (Wendel 1898). Therefore, GV can be ruled out from the differential diagnosis list in this case. Additionally, we considered whether the observed GBA in this case could be attributed to GBI (Olivares et al. 2018). However, in cases of GBI, bile leakage occurs as a result of gallbladder rupture. This condition is characterised by severe abdominal pain and can lead to peritonitis, indicating a critical situation for the patient. In contrast, the imaging examination of the patient in this case revealed no signs of ascites or peritonitis, despite presenting symptoms of pancreatitis. These findings at least rule out the possibility that GBA resulted from rupture due to infarction.

This case report has some limitations that need to be addressed. The abdominal ultrasound conducted during the patient’s initial visit did not involve a thorough evaluation of the pancreas. Consequently, the assessment regarding pancreatitis relied on the results of the cPL test and CT scan images. At the time of the initial visit, the patient exhibited poor cooperation during the acquisition of abdominal ultrasound images, particularly when imaging the gallbladder. To visualise the gallbladder, the probe’s position was adjusted from various angles, and obtaining images in a modified transverse plane was challenging.

Additionally, the hyperechoic nodule suspected to be a gallbladder stone appears to be located in the centre of the gallbladder in the image; however, due to the patient’s lack of cooperation and the positioning of the modified probe during image acquisition, this observation lacks clarity. It is presumed that the unclear visualisation of the gallbladder wall may contribute to this issue. Despite challenges in performing an abdominal ultrasound, the high concentration of cPL, the discovery of a hyperattenuated nodule in the upper abdominal gallbladder, and the evidence of atrophic gallbladder observed in a repeated CT image after 4 years align with the limitations mentioned earlier. Nevertheless, there is a significant possibility of GBA related to pancreatitis in this case.

This case report presents an occurrence of spontaneous GBA observed over a 4-year period in a dog diagnosed with pancreatitis and exhibited mild digestive symptoms. Notably, the GBA occurred without undergoing cholecystectomy. To the best of the authors’ knowledge, while GBA is rarely reported in human medicine according to available literature, this case represents presumably the first reported occurrence of GBA in veterinary medicine. There have been reports of GBA associated with pancreatitis in humans (Jiang et al. 2023); it is postulated that a similar progression occurred in dogs, as in this case. While histopathological examination of the atrophic gallbladder would provide the highest diagnostic value, a biopsy was not performed following detailed discussions with the patient’s owner. Despite the absence of a biopsy, we present this case of GBA on the basis of clinical and radiographic follow-up evidence.

In conclusion, this is a case of GBA caused by chronic gallbladder irritation due to pancreatitis, and it is an extremely rare case that has, to our knowledge, not yet been reported in the veterinary literature. To elucidate the pathogenesis of GBA in dogs, more research is needed.

## References

[R1] Aguirre A. Diseases of the gallbladder and extrahepatic biliary system. In: Ettinger SJ, Feldman EC, Cote E, editors. Textbook of veterinary internal medicine. St. Louis, USA: Elsevier; 2017. p. 1674-80.

[R2] Bartlett JR, Friedman KJ, Ling SC, Pace RG, Bell SC, Bourke B, Castaldo G, Castellani C, Cipolli M, Colombo C, Colombo JL, Debray D, Fernandez A, Lacaille F, Macek M Jr, Rowland M, Salvatore F, Taylor CJ, Wainwright C, Wilschanski M, Zemkova D, Hannah WB, Phillips MJ, Corey M, Zielenski J, Dorfman R, Wang Y, Zou F, Silverman LM, Drumm ML, Wright FA, Lange EM, Durie PR, Knowles MR; Gene Modifier Study Group. Genetic modifiers of liver disease in cystic fibrosis. JAMA. 2009 Sep 9;302(10):1076-83. 19738092 10.1001/jama.2009.1295 PMC3711243

[R3] Center SA, Slater MR, Manwarren T, Prymak K. Diagnostic efficacy of serum alkaline phosphatase and gamma-glutamyltransferase in dogs with histologically confirmed hepatobiliary disease: 270 cases (1980–1990). J Am Vet Med Assoc. 1992 Oct 15;201(8):1258-64. 1358870

[R4] Chen JH, Stoltz DA, Karp PH, Ernst SE, Pezzulo AA, Moninger TO, Rector MV, Reznikov LR, Launspach JL, Chaloner K, Zabner J, Welsh MJ. Loss of anion transport without increased sodium absorption characterizes newborn porcine cystic fibrosis airway epithelia. Cell. 2010 Dec 10;143(6):911-23. 21145458 10.1016/j.cell.2010.11.029 PMC3057187

[R5] Hardy J, Margolis JJ, Contag CH. Induced biliary excretion of Listeria monocytogenes. Infect Immun. 2006 Mar;74(3):1819-27. 16495556 10.1128/IAI.74.3.1819-1827.2006 PMC1418634

[R6] Hatakeyama K, Nagakawa T, Suga T, Miyakawa H, Hirayama A, Matsunaga T, Okamura K, Suzuki H, Honma S, Okada K, Iwaguchi T, Muraoka S. [Carcinoma of the gallbladder which progress on the mucosa of choledochocolonic fistula]. Nihon Shokakibyo Gakkai Zasshi. 2009 Jul;106(7):1063-9. Japanese. 19578315

[R7] Hofmann AF. The continuing importance of bile acids in liver and intestinal disease. Arch Intern Med. 1999 Dec;159(22):2647-58. 10597755 10.1001/archinte.159.22.2647

[R8] Jiang F, Zhang J, Hu Z. Risk factors for pancreatitis occurrence after gallstone treatment using endoscopic retrograde cholangiopancreatography. Afri Health Sci. 2023;23(2):231-8. 10.4314/ahs.v23i2.26 PMC10782312 38223651

[R9] Kim WR, Flamm SL, Di Bisceglie AM, Bodenheimer HC; Public Policy Committee of the American Association for the Study of Liver Disease. Serum activity of alanine aminotransferase (ALT) as an indicator of health and disease. Hepatology. 2008 Apr;47(4):1363-70. 18366115 10.1002/hep.22109

[R10] King LJ, Scurr ED, Murugan N, Williams SG, Westaby D, Healy JC. Hepatobiliary and pancreatic manifestations of cystic fibrosis: MR imaging appearances. Radiographics. 2000 May-Jun;20(3):767-77. 10835127 10.1148/radiographics.20.3.g00ma08767

[R11] Kramer NR, Karasick D, Karasick S. “Micro-gallbladder” – A clue to cystic fibrosis. J Can Assoc Radiol. 1983 Dec;34(4):271-2. 6668284

[R12] Lee HW, Osis G, Handlogten ME, Guo H, Verlander JW, Weiner ID. Effect of dietary protein restriction on renal ammonia metabolism. Am J Physiol Renal Physiol. 2015 Jun 15;308(12):F1463-73. 10.1152/ajprenal.00077.2015 PMC4469882 25925252

[R13] Luo P, Wang C, Zhang G. A rare case report of chronic cholecystitis complicated with incomplete gallbladder volvulus. Int J Clin Exp Med. 2014 Oct 15;7(10):3602-4. 25419406 PMC4238524

[R14] Maldonado CZ, Ruiz Lopez MJ, Gonzalez Valverde FM, Alarcon Soldevilla F, Pastor Quirante F, Garcia Medina V. Ultrasound findings associated to gallbladder carcinoma. Cir Esp. 2014 May;92(5):348-55. 24629915 10.1016/j.ciresp.2012.10.007

[R15] McGill MR. The past and present of serum aminotransferases and the future of liver injury biomarkers. EXCLI J. 2016 Dec 15;15:817-28. 28337112 10.17179/excli2016-800 PMC5318690

[R16] Olivares G, Fernandez Y, Di Palma S, Murgia D. Spontaneous gall bladder infarction in a dog with a congenital extrahepatic portosystemic shunt. Vet Rec Case Rep. 2018;6(1):e000557.

[R17] Pak M, Lindseth G. Risk factors for cholelithiasis. Gastroenterol Nurs. 2016 Jul-Aug;39(4):297-309. 27467059 10.1097/SGA.0000000000000235 PMC8802735

[R18] Satoh H, Harada M, Tashiro S, Shiroya T, Imawaka H, Machii K. The effect of continuous arterial infusion of gabexate mesilate (FOY-007) on experimental acute pancreatitis. J Med Invest. 2004 Aug;51(3-4):186-93. 15460905 10.2152/jmi.51.186

[R19] Shirah BH, Shirah HA, Albeladi KB. Mirizzi syndrome: Necessity for safe approach in dealing with diagnostic and treatment challenges. Ann Hepatobiliary Pancreat Surg. 2017 Aug;21(3):122-30. 28989998 10.14701/ahbps.2017.21.3.122 PMC5620472

[R20] Siddique A, Kowdley KV. Approach to a patient with elevated serum alkaline phosphatase. Clin Liver Dis. 2012 May;16(2):199-229. 22541695 10.1016/j.cld.2012.03.012 PMC3341633

[R21] Simpson KW. Pancreatitis and triaditis in cats: Causes and treatment. J Small Anim Pract. 2015 Jan;56(1):40-9. 25586805 10.1111/jsap.12313

[R22] Spadafora D, Hawkins EC, Murphy KE, Clark LA, Ballard ST. Naturally occurring mutations in the canine CFTR gene. Physiol Genomics. 2010 Aug;42(3):480-5. 20571109 10.1152/physiolgenomics.00092.2010 PMC2929888

[R23] Taboada J, Meyer DJ. Cholestasis associated with extrahepatic bacterial infection in five dogs. J Vet Intern Med. 1989 Oct-Dec;3(4):216-21. 2585368 10.1111/j.1939-1676.1989.tb00860.x

[R24] Wendel AV. VI. A case of floating gall-bladder and kidney complicated by cholelithiasis, with perforation of the gall-bladder. Ann Surg. 1898 Feb;27(2):199-202. PMC1426674 17860545

